# Enhancing disaster response through comprehensive transportation models: insights from the Kahramanmaraş earthquakes

**DOI:** 10.1186/s13049-023-01113-w

**Published:** 2023-09-14

**Authors:** Ali Cankut Tatliparmak, Rohat Ak

**Affiliations:** 1 Faculty of Medicine, Dept. of Emergency Medicine, Uskudar University, Site Yolu Cd No:27, 34768 Ümraniye, Istanbul, Turkey; 2 Dept. of Emergency Medicine, University of Health Sciences, Kartal Dr. Lutfi Kirdar City Hospital, 47 Cevizli Mevkii, Kartal, İstanbul, 34865 Turkey

**To the editor**,

We commend Dr. Yilmaz for his insightful commentary titled “Transportation Model Utilized in the First Week following the Kahramanmaraş Earthquakes in Turkey - Transport Health Centers” [1]. While his effort to elucidate the transportation model utilized during the earthquakes is noteworthy, we believe that certain facets of the commentary’s depth could benefit from further enhancement.

The commentary adeptly portrays the consequences of the earthquake—loss of life, infrastructure damage, and healthcare strain. Turkey’s strategic actions, involving the deployment of healthcare professionals, essential equipment, and Transport Health Centers, highlight a coordinated approach for comprehensive mitigation. Detailed transportation measures—such as healthcare worker mobilization, medical equipment logistics, and patient evacuation—reveal the intricacies of the response. The author emphasizes preparedness for future calamities, underscoring evolving transportation models that address natural disaster challenges. This serves as a poignant reminder of the sustained commitment required to enhance global disaster response capabilities.

However, the commentary’s treatment of the challenges and limitations that emerged during the model’s implementation lacks depth. While acknowledging disruptions in road traffic due to seismic impact and adverse weather conditions, further elaboration on the nuanced strategies deployed to surmount these impediments would enhance the discourse. Additionally, the commentary warrants a more intricate exploration of the coordination and collaboration dynamics among diverse stakeholders involved in the transportation model. Considering the scale of the disaster and the multitude of entities involved, conducting a comprehensive scrutiny of coordination mechanisms and potential challenges during the transportation process would offer a more holistic perspective.

Furthermore, the commentary briefly acknowledges the establishment of Transport Health Centers for treating injured patients but omits a comprehensive evaluation of their efficacy. Including data encompassing center capacity, resource allocation, care quality, lessons learned, and potential enhancements would enhance comprehension of their role in forthcoming disaster responses.

Moreover, a notable gap in the article is the absence of specifics regarding the transportation of critical medical supplies, particularly temperature-sensitive and delicate items such as blood and vaccines, as well as the cost-effectiveness of this implementation. Given the imperative of effective disaster management, incorporating these details could potentially amplify the applicability of this transportation model in forthcoming disaster scenarios [2].

Additionally, the commentary overlooks the opportunity to delve into the comparative advantages of the discussed transportation model in comparison to established paradigms within the literature. Exploring these distinctions could provide valuable insights for readers seeking to ascertain the model’s distinctiveness and utility [3, 4].

We anticipate that future discussions on this subject will encompass more comprehensive analyses and evaluations of transportation models employed in the context of natural disasters.

## Data Availability

Not applicable.
